# Natural distribution of environmental radon daughters in the different brain areas of an Alzheimer Disease victim

**DOI:** 10.1186/1750-1326-1-11

**Published:** 2006-09-11

**Authors:** Berislav Momčilović, Glenn I Lykken, Marvin Cooley

**Affiliations:** 1 Institute for Medical Research and Occupational Health, PO Box 291, 10001 Zagreb, Croatia; 2 Department of Physics, University of North Dakota, Grand Forks, ND 58202-7129, USA; 3 Department of Pathology, University of North Dakota, Grand Forks, ND 58202-7129, USA

## Abstract

**Background:**

Radon is a ubiquitous noble gas in the environment and a primary source of harmful radiation exposure for humans; it decays in a cascade of daughters (RAD) by releasing the cell damaging high energy alpha particles.

**Results:**

We studied natural distribution of RAD ^210^Po and ^210^Bi in the different parts of the postmortem brain of 86-year-old woman who had suffered from Alzheimer's disease (AD). A distinct brain map emerged, since RAD distribution was different among the analyzed brain areas. The highest RAD irradiation (mSv·year^-1^) occurred in the decreasing order of magnitude: amygdale (Amy) >> hippocampus (Hip) > temporal lobe (Tem) ~ frontal lobe (Fro) > occipital lobe (Occ) ~ parietal lobe (Par) > substantia nigra (SN) >> locus ceruleus (LC) ~ nucleus basalis (NB); generally more RAD accumulated in the proteins than lipids of gray and white (gray > white) brain matter. Amy and Hip are particularly vulnerable brain structure targets to significant RAD internal radiation damage in AD (5.98 and 1.82 mSv·year^-1^, respectively). Next, naturally occurring RAD radiation for Tem and Fro, then Occ and Par, and SN was an order of magnitude higher than that in LC and NB; the later was within RAD we observed previously in the healthy control brains.

**Conclusion:**

Naturally occurring environmental RAD exposure may dramatically enhance AD deterioration by selectively targeting brain areas of emotions (Amy) and memory (Hip).

## Background

Alzheimer's Disease (AD), the most common cause of dementia in the elderly, is a progressive neurodegenerative disease of unknown origin that gradually robs the patient of cognitive function and eventually causes death [1]. Recently, we showed that radon daughters ^210^Po and ^210^Bi (RAD), accrue selectively in the brain proteins and lipids of men and women who suffered from AD and Parkinson's Disease, respectively [2,3]. We proposed that AD is a systemic brain-cell disease which selectively involves the cell membrane protein structures of ion gates, pores, and channels, with consequent chlorine leaking into the cells, collapse of the cell membrane gradient, and functional cell death. Other authors proposed calcium and potassium channel impairment, respectively [4,5]; the latter authors proposed AD to be a general systemic disease of the body as somatic fibroblasts showed the same Ca-channel defect as that of neurons. Thus, the pathological substrate of AD may well be described as "channelopathy", a condition where the impaired cell membrane protein structures lead to the deregulation of the ionic influx by the brain cells [6-9].

Most of the AD studies are limited to the brain cortex and limbic system, notably the hippocampus, since these are the well recognized brain areas involved in the AD memory loss [10]; the role of other brain structures in AD is poorly understood [11-13]. In this case report, we studied the distribution of naturally occurring environmental RAD in different brain areas in AD. Radon is a ubiquitous noble gas in the air we breath [14], it is lipid soluble and (in spite of being a noble gas) capable of forming weak chemical bonds [15], and tends to accumulate in high-carbon body fat tissue including the brain [16]. Radon and RAD are the source of four cell destructive high energy alpha particles which may significantly contribute to the internal radiation dose of the brain and play a role in AD etiology and pathology [17,18].

## Subject and methods

We studied the distribution of naturally occurring environmental RAD polonium-210 (^210^Po; alpha particle emitter) and bismuth-210 (^210^Bi; beta particle emitter) in nine different brain regions of an 86-year-old deceased woman. She was a resident of North Dakota who suffered from AD at old age and with otherwise uneventful medical history. Post-mortem samples were obtained from all four brain lobes, i.e., frontal (Fro). parietal (Par), occipital (Occ), and temporal (Tem), and from the five well defined inside the brain structures, i.e., the hippocampus (Hip), amygdale (Amy), substantia nigra (SN), locus ceruleus (LC), and nucleus basalis (NB). The pathological diagnosis of AD was based on the presence of an age-adjusted moderate to a severe number of plaques in the neocortex [19]. We separated cortical gray and subcortical white matter from each brain lobe and separate gray and adjacent white matter for every subcortical ganglion. One gram of each sample was fractionated into protein and lipid content before assessing ^210^Bi and ^210^Po activity separately.

Quantitative determination of the proteins and lipids from selected brain regions and their ^210^Po and ^210^Bi radioactivity was performed as described previously [2,3]. In brief, the proteins and lipids were extracted from the gray and white matter of the brain and ganglia by following the respective methods of Bradford [20] and Folch et al. [21]. The protein fraction was passed through a polymembrane under a negative pressure gradient [22]. The ^210^Po from the samples and ^208^Po from a spiked solution were plated on a silver disc [23]. Alpha and beta particle activities were determined in an Alpha Spectrometer System supplied with a radionuclide library software package (EG&G ORTEC, Oak Ridge, TN) and a Beckman scintillation spectrometer (Beckman Co., Fullerton, CA), respectively.

Lead-210 (^210^Pb) decays to ^210^Bi, which in turn decays to ^210^Po; each decays at a different rate. After 600 days (1.64 years) a "secular equilibrium" is reached; the activities of the ^210^Bi and ^210^Po in the sample are then equal to the ^210^Pb activity. The standard Bateman differential equation of growth and decay of radio-nuclides in a decay chain was used to correct for ^210^Po formation from ^210^Bi directly and from ^210^Pb indirectly via ^210^Bi [24].

The radioactivity of ^210^Bi and ^210^Po was assessed in replicates of every studied brain area and expressed in μBq·g^-1 ^tissue (1 μBq equals 1 disintegration per 10^6 ^second, i.e., 31 disintegrations per year). The difference between the two replicates of the same sample and for the same radionuclide didn't exceed 5%. We consider the difference in the RAD radionuclide retention between any two brain areas of >20% to be significant (>>), that of 10–20% to be probably significant (>), and that below 10% as non-significant (~). The normal range of the biological variability is ± 20%, and the maximal acceptable difference between the ^210 ^Po and ^210^Bi in the same brain area sample was set at 10%.

**Figure 1:**
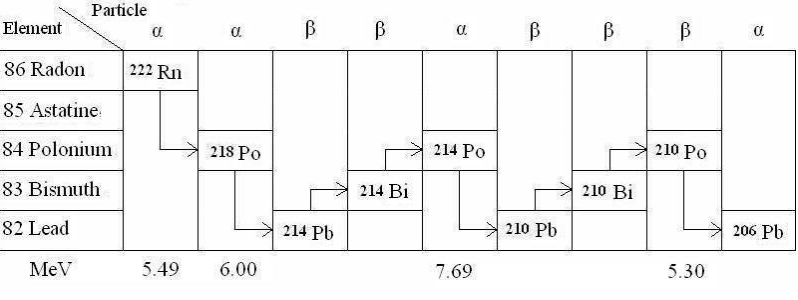
Radon (^222^Rn) radiation decay.

The particular brain area cell death radiation risk to the high energy RAD alpha particles was calculated on the assumption of the brain cell density of 6.4·10^6^·g^-1 ^(90% glia, 10% neurons) and the average weight of the adult female brain of 1250 g [25,26]; the brain weight of our subject was in that category. The absorbed physical energy was first expressed in micro Grays (μGy) and then transformed to mili Sieverts (mSi) to provide for the assessment of the biological effective dose of radiation [27]. It should be noted that every single ^210^Po disintegration means an instant death to a minimum of three cells along the path of its high energy (5.305MeV) decaying alpha particle [28]; altogether, there are four such "killing" alpha particles in the radon decay chain (Fig 1)[15].

This research was approved by the University of North Dakota Institutional Review Board, Grand Forks, ND (IRB-9509-027), and carried out in full compliance with Helsinki Declaration.

## Results

**Figure 2:**
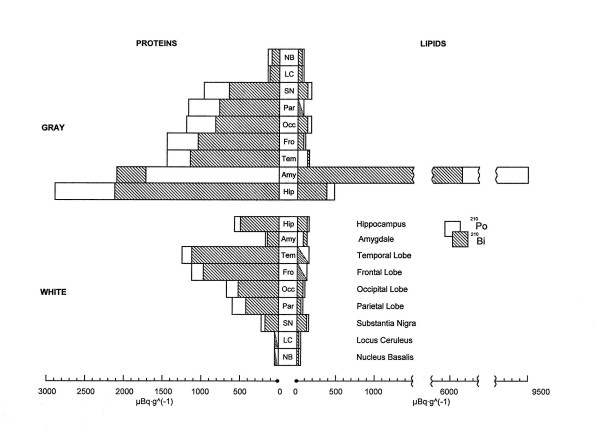
Brain structure distribution of polonium-210 (□) and bismuth-210 (■) in the proteins and lipids of the gray and white brain matter in an Alzheimer disease victim (μBq g^-1 ^tissue).

The results showed a highly selective distribution of ^210^Po and ^210^Bi in the proteins and lipids from the gray and white matter of the brain and ganglia in AD; indeed, RAD accumulation differs significantly between the brain areas (Fig 2). We observed a very good congruence of ^210^Po and ^210^Bi in all the duplicate samples from the same brain areas, indicating reliability of the results obtained by the two different analytical methods of alpha and beta particle counting; values for ^210^Po tend to be somewhat higher than that of ^210^Bi but still within the accepted limit of accuracy. As a rule, RAD accumulation was higher in the proteins than lipids of various brain structures, and higher in the gray than white brain matter proteins, respectively.

The retention of RAD in the gray brain matter proteins was, in the decreasing order of magnitude:

Hip >> Amy >> Tem ~ Fro > Occ ~ Par > SN >> LC ~ NB.     (A)

RAD retention was generally lower in the white matter brain and cerebral ganglia proteins than that of the gray brain matter. The comparable sequence for both RAD (^210^Po and ^210^Bi) in the white brain matter proteins was somewhat different from that in the grey matter:

Tem > Fro>> Occ ~ Par ~ Hip >> SN > Amy >> LC ~ NB     (B)

It should be noted that the range of the ^210^Po activity in the gray matter proteins of the different brain structures (A) may be as low as 150 μBq for NB and as high as 2906 μBq in the Hip [or 100 vs. 2138 μBq (per gram) of ^210^Bi for the same brain structures], a factor of 20 difference! A similar range was also observed in the proteins from the brain white matter (B), although the actual sequence was somewhat different (50 vs. 1152 μBq·g^-1^of ^210^Po and 50 vs. 1119 μBq·g^-1^of ^210^Bi for NB and Tem, respectively).

The notable exception to the uniformly higher RAD accumulation in the gray (A) and white (B) brain matter proteins than lipids in AD was the accentuated RAD retention in the amygdale lipids. Indeed, the retention of ^210^Po and ^210^Bi in the Amy lipids reached astonishing 9285 and 6162 μBq·g^-1^, respectively, well above anything we have observed of RAD in any other brain area. The retention of 468 μBq·g^-1 ^of ^210^Po and 362 μBq·g^-1 ^of ^210^Bi in the Hip was the next highest for the RAD retention in the lipids. Although increased in relation to the other lipid RAD, the Hip lipid RAD retention was at the lower end of the RAD activities seen in the proteins.

To better assess the radiation risk from ^210^Po "killer" alpha particles over the last year of the subject's life, we combined together ^210^Po activity in the gray brain matter proteins and lipids, since proteins and lipids from the same anatomical structure are not naturally separated. Apparently, a substantial brain cell loss should have occurred in the Amy and Hip as a result of the cell killing potential of the high energy RAD alpha particles (Table 1) [29]. The decreasing order of magnitude sequence of RAD ^210^Po in proteins and lipids from different brain areas showed the radiation risk to be:

**Table 1:** Estimated annual regional brain cell loss and cell dose per gram tissue from ^210^Po high energy alpha particles (5.305 MeV) in the proteins and lipids.*

Brain structure	Emissions	Estimated cell loss		Cell dosimetry Energy absorbed	Biolgical effectiveness
	Bq•y•g^-1^	Number	%•y^-1^	βGy•yr-^1^	mSv•y^-1^
Amygdale	347 828	1 043 484	16.65	295	5.90
Hippocampus	106 475	319 426	4.99	90	1.80
Temporal lobe	50 078	150 233	2.35	42	0.85 0.79^a^
Frontal lobe	49 545	148 635	2.32	42	0.84 0.75^a^
Occipital lobe	43 739	131 217	2.05	37	0.74
Parietal lobe	39 005	117 016	1.83	33	0.66
Substantia nigra	30 855	92 564	1.45	27	0.54
Locus ceruleus	6 848	20 544	0.30	6	0.12
Nucleus basalis	6 848	20 544	0.30	6	0.12

*The average person absorbs ≈450 βGy of cosmic radiation in a year. Since all ^210^Po radiation is composed solely of alpha particles then a relative effective factor is 20 (1 Sv = 20 Gy) [29].

^a^Momčilović et al.[2,3].

Amy >> Hip > Tem ~ Fro > Occ ~ Par > SN >> LC ~ NB     (C)

We estimated that a minimum of 15% of amygdale cell population per gram tissue (>10^6 ^cells) was destroyed over a single year, about 5% of hippocampus cell population was also destroyed over the same time, and as much as 1–2% of that in the four brain lobe cell population. Since the brain lobes have a total cell mass considerably greater than that of Amy and Hip, the actual cell loss from brain lobes would be also substantial. Evidently, the different areas of the AD brain are exposed to a different radiation risk and consequent cell loss.

## Discussion

The major finding of this case report is that the explored areas of the AD brain are specifically and selectively targeted by RAD, so that there is a different radiation risk to the various brain structures at the same environmental radon and RAD exposure. It has never before been observed that naturally occurring environmental RAD can reach dangerous levels of radiation exposure in certain brain areas such as Amy and Hip. These findings also confirmed our previous observations about brain proteins as a targeted biochemical compartment in the AD brain. Indeed, the RAD deposition in the gray and white brain matter proteins and lipids from the frontal and temporal brain lobes of this single subject were within the average values we reported for the same brain structures in a group of people who suffered from AD (see Table 1) [2,3]. Consequently, if the RAD deposition to the proteins and lipids of the two identical brain regions in these two separate studies is approximately equal, it is reasonable to assume that RAD distribution in all the other areas of the AD brain would also have the same pattern of distribution as found here. North Dakota is known for it's high RAD [31], but the RAD in the frontal and temporal region of this case is very well within the average RAD from the same brain region samples obtained from the Alzheimer's Foundation [2,3]. Since the Alzheimer's Foundation brains came from the different parts of the USA, it appears that, according to our instrument limits, the regional environmental exposure to RAD did not affect the RAD brain distribution.

Our study identified the proteins in the hippocampus and amygdale as a two primary brain area targets for RAD in AD. Further, we noted that there was two times more RAD deposited in hippocampus than in the cortex per unit mass of the protein. This is an indicative ratio since the Hip is assembled of three cellular layers identical with three out of six cell layers of the brain cortex [25]. Thus, if three Hip layers yield two times more RAD than the six layers of the brain cortex, and three of the cortex layers are identical with those of Hip, it is evident that the presence of three more (but different) cortical layers did not contributed to the RAD; therefore we think that our finding supports the concept of laminar specificity of cortical pathology in AD [10] We also noted biochemical similarity of RAD retention in the parietal and occipital lobe proteins vs. that in respective frontal and temporal lobes; the later was higher (C).

The lowest RAD retention was observed in the proteins of locus ceruleus and nucleus basalis, otherwise an area where a great neuronal loss was reported for LC, NB, and SN in AD subjects [13,31]. Since neurons are also composed of proteins, this observation implies how proteins from these brain areas may have either different affinity for RAD or, perhaps, the fact that we analyzed proteins in both neurons and glia cells of the brain; the later is much more abundant (90%). Events like impaired conformational changes in protein post-synaptic scaffolding [32], the fall in number of neuron synaptic contacts [33] the failing support of astrocytes which are especially vulnerable to the ionic radiation [34], and cyto-architectonic collapse of functional neurons [35], may all precede the neuronal loss in AD brain.

We predicted correctly that, as in the previous AD study, RAD would as well selectively accrue in the brain cell proteins of Amy and Hip [2,3]; what we didn't know at that time was that different brain areas would had quite distinct RAD affinity. Essentially, our finding of high protein RAD affinity in AD credits the importance of *mal *variant AD proteins in the neurons [6-8], with a caveat that actual *mal *variant protein biochemical structure may be quite different for various brain areas and their cell population. The reason for the repeatedly observed high RAD protein affinity and respective brain area specific protein affinity in AD remains obscure. We only know that the biochemical structure of the AD protein could be changed such that more carbon bonds would be available to moderate radon movement before radon decays in RAD; it may be even some variety of a prion protein of a chronic disease [9]. It has been shown that metals Al, Cu, Fe, Pb, Si, and Zn acts like a potent "seeding" factors inducing excessive amyloid Aβ peptide formation [36-39]. Indeed, Aβ is a major protein component of the senile plaque and what is the hallmark of AD [40]; the degradation of these excess proteins in the AD brain is further reduced by the lack of the proteosomes, a large protein complex responsible for intracellular degradation of misfolded, oxidized, or aggregated proteins [41]. Since radon is a radioactive noble gas it transfers freely across the blood-brain barrier in and out of the brain; when radon decays to RAD both the high energy of cell killing potential is released and the heavy metals generated, the later would act as a potent seeding agents for Aβ generation. Thus, radon can be both a direct cause of AD via the imunogenic debris of the killed cells of already changed proteins in the AD brain, and by also enhancing Aβ synthesis.

The only exception to the rule that AD specifically targets the brain matter proteins was that in this subject the highest RAD deposition was observed in the amygdale gray matter lipids. Since we already saw selective RAD accumulation to the brain lipids in Parkinson's disease [2,3], we concluded that an unfortunate event had occurred to this study's subject. i.e., the combined protein and lipid cell membrane chanellopathy. This failure of AD brain cell lipids may be secondary to the failures of protein folding, their conformation change, appearance of false ionic channels, and consequent failure in the Ca-channel ionic cell influx [3,42,43].

We are impressed that the tiny amygdale alone received an equivalent of 2/3 of the respective total yearly human body physical energy dose from cosmic rays (299 vs. 450 μG·y^-1^) [29]. The biological quality of different types of radiation is different (alpha being the most adverse to the biological tissue), and when the results are corrected for the high biological quality factor of alpha radiation, Amy will receive a fifteen-times greater biological effective radiation dose than the whole human body over the entire year (5980 vs. 450 μSi·y^-1^, respectively). Approximately one million of Amy cells will be killed in a year, an equivalent of one gram of that 10 gram heavy brain structure. This estimate is a very conservative approximation, based on only three direct cell kills per decayed high energy alpha particle, since as much as fifty cells may be irreparably injured and die after some delay by a single ^210^Po alpha particle as a result of the "bystander effect" [44]. What we observed in this AD brain is the internal radiation "amygdalectomy" and how that might explain some of the respective emotional torpidity and insensitivity associated with AD and old age. This case appears to fit the usual pattern of events in AD; on average, person spend several years in the mild or minimal stages, between 4 and 5 years in the moderate disease stages, and depending on the quality of care in the depending stages, a year or more requiring full nursing care [45].

## Conclusion

In conclusion, AD is a complex and progressive brain disease characterized by the failing ability to cope with environmental xenobiotic hazards [2,3], excessive free radical injury, inflammation and immunity deficiency [46], cell repair impairment [47], and the protein synthesis [48]. The ubiquitous environmental RAD exposure, and high RAD accumulation in the sensitive brain structures may either induce or hasten or both the irreversible "shut down" process of the ailing human brain in AD.

## Competing interests

The author(s) declare that they have no competing interests.

## Authors' contributions

BM planned and designed the study, did data analysis and interpretation, and drafted the manuscript. GIL conceived the study, coordinated the radio analytical work, did dose calculations and helped to draft the manuscript. MC did pathology, pathological diagnosis, disease classification, brain dissection, and helped towards clinical data interpretation.
